# Multicenter Validation of Foundation Model Adaptation for Automated Pancreatic Tumor Delineation on CT Scans

**DOI:** 10.3390/cancers18172836

**Published:** 2026-09-01

**Authors:** Halil Ertugrul Aktas, Eminenur Sen Tasci, Linkai Peng, Muhammed Enes Tasci, Yavuz B. Taktak, Sitki Safa Taflan, Baver Tutun, Fergan Bol, Murat Iren, Mucahit Ekici, Andrea Mia Bejar, Elif Keles, Hongyi Pan, Wanying Dou, Burak Gultekin, Alper Akin, Oyku Ikizgul, Okan Cetin, Emre Uysal, Maide Mureva, Mustafa Orhan Nalbant, Nurullah Kaya, Alpay Medetalibeyoglu, Kadir Atakir, Berna Akkus Yildirim, Gulbiz Dagoglu Kartal, Zongwei Zhou, Sukru Mehmet Erturk, Frank H. Miller, Gorkem Durak, Ulas Bagci

**Affiliations:** 1 Machine & Hybrid Intelligence Lab, Department of Radiology, Northwestern University, Chicago, IL 60611, USA; 2 Department of Radiology, Istanbul Faculty of Medicine, Istanbul University, Istanbul 34093, Turkey; 3 Department of Radiation Oncology, University of Health Sciences, Prof. Dr. Cemil Tascioglu City Hospital, Istanbul 34384, Turkey; 4 Department of Radiology, University of Health Sciences, Istanbul Bakirkoy Dr. Sadi Konuk Training and Research Hospital, Istanbul 34147, Turkey; 5 Department of Radiology, Harran University, Sanliurfa 63300, Turkey; 6 Department of Internal Medicine, Uskudar State Hospital, Istanbul 34662, Turkey; 7 Department of Internal Medicine, Istanbul Faculty of Medicine, Istanbul University, Istanbul 34093, Turkey; 8 Department of Medicine, Boston Medical Center—Brighton, Boston University Chobanian & Avedisian School of Medicine, Boston, MA 02135, USA; 9 Department of Radiology, University of Chicago, Chicago, IL 60637, USA; 10 Department of Radiology, University of Health Sciences, Prof. Dr. Cemil Tascioglu City Hospital, Istanbul 34384, Turkey; 11 Department of Computer Science, Johns Hopkins University, Baltimore, MD 21218, USA

**Keywords:** pancreatic ductal adenocarcinoma, pancreatic tumor segmentation, foundation models, Segment Anything Model, task-specific adaptation, fine-tuning, contrast-enhanced computed tomography

## Abstract

**Background**: Accurate pancreatic tumor segmentation on contrast-enhanced computed tomography (CECT) is important for staging, treatment planning, and response assessment in pancreatic ductal adenocarcinoma (PDAC). Although foundation models have shown promise for medical image segmentation, their effectiveness for disease-specific tumor delineation remains uncertain. This study evaluated whether fine-tuning a Segment Anything Model (SAM)-based framework on the target cohorts improves pancreatic tumor segmentation compared with directly applied foundation models. **Methods**: In this retrospective multicenter study, CECT examinations from patients with pathologically confirmed PDAC acquired between 2015 and 2025 were included. Two foundation model baselines, nnInteractive and MedSAM2, were compared with three TAGS-based configurations representing increasing levels of adaptation: TAGS (Zero-Shot), SAM-TAGS (fine-tuned from SAM weights), and MSD-TAGS (fine-tuned from a pancreas-specific checkpoint). Performance was assessed using patient-level five-fold cross-validation with the Dice Similarity Coefficient (DSC) and Normalized Surface Dice (NSD). A leave-one-center-out analysis was additionally performed to evaluate generalization to institutions absent from training. **Results**: A total of 500 patients with PDAC from four independent centers were included. MSD-TAGS achieved the highest overall segmentation performance, with a mean DSC of 0.703 and a mean NSD of 0.835. SAM-TAGS achieved a mean DSC of 0.691 and a mean NSD of 0.822. In comparison, nnInteractive achieved a mean DSC of 0.632 and a mean NSD of 0.782; MedSAM2, 0.585 and 0.792; and TAGS (Zero-Shot), 0.496 and 0.639, respectively. Compared with each competing method, MSD-TAGS achieved significantly higher DSC and NSD values (all Holm-adjusted *p* < 0.001). It also achieved the highest DSC across all four centers and the highest NSD in three of the four centers. Under leave-one-center-out evaluation, MSD-TAGS declined by 0.025 DSC and SAM-TAGS by 0.089. **Conclusions**: Task-specific adaptation improved pancreatic tumor segmentation with foundation models, and fine-tuned TAGS outperformed two publicly released promptable models applied without adaptation. Models initialized from a pancreas-specific checkpoint showed greater robustness under center-held-out evaluation. These findings support the importance of organ-specific initialization and target-cohort fine-tuning for disease-specific segmentation and warrant further validation before clinical translation. The multicenter pancreatic tumor CT segmentation dataset, including de-identified images and expert segmentation annotations, is publicly available to facilitate future research.

## Simple Summary

Pancreatic cancer is one of the deadliest cancers, and accurate tumor delineation on contrast-enhanced computed tomography (CECT) is essential for staging, treatment planning, and response assessment. Manual tumor segmentation is time-consuming and subject to reader variability. Recently, foundation models have shown promise for medical image segmentation, but their effectiveness for disease-specific tasks such as pancreatic tumor delineation remains uncertain. In this multicenter study, we extended our previously developed Tumor-Adaptive Guidance for SAM (TAGS) framework to a large multicenter cohort and benchmarked it against foundation model-based segmentation approaches. Using 500 CECT examinations from four institutions, we found that fine-tuned TAGS variants outperformed two off-the-shelf foundation models applied without adaptation. We additionally release a multicenter pancreatic tumor CT segmentation dataset to facilitate future research and benchmarking.

## 1. Introduction

Pancreatic ductal adenocarcinoma (PDAC) remains one of the most lethal malignancies worldwide, characterized by clinically silent progression, late detection, and poor survival outcomes [1]. At diagnosis, only 15.4% of patients present with localized disease, whereas approximately 51% present with distant-stage disease. This distribution translates into markedly different outcomes, with five-year relative survival decreasing from 43.6% for localized disease to 3.4% for distant-stage disease [2]. This predominance of advanced-stage presentation contributes to the persistently low overall five-year relative survival rate (13.7%) and limits the proportion of patients eligible for potentially curative surgical resection [2,3]. Therefore, effective management depends on accurate staging, treatment selection, and timely assessment of progression or response, all of which rely heavily on imaging. However, current imaging workflows struggle to reliably characterize local tumor extent and vascular involvement [4,5], key features that directly inform resectability classification and surgical decision-making [4,5,6].

Contrast-enhanced computed tomography (CECT) is the primary imaging modality for the initial evaluation and staging of suspected PDAC [7]. However, radiologic assessment remains challenging because tumor conspicuity, boundary definition, and vascular involvement vary widely depending on lesion size, anatomical location, and imaging phenotype [8,9]. Objective pancreas and tumor segmentation could help address these challenges by supporting quantitative assessments of tumor burden, spatial localization, tumor–vessel interface, and longitudinal evaluation during follow-up [10,11]. Despite this potential, manual segmentation remains highly time-consuming and operator-dependent in clinical practice [12], limiting its scalability for large imaging cohorts and routine workflows.

To address these challenges, artificial intelligence (AI)-based segmentation methods have been introduced to automate and standardize tumor delineation. Among these approaches, conventional supervised models often require large annotated datasets and involve resource-intensive development [13,14]. More recently, foundation models have offered a different approach by combining large-scale pretraining with prompt-based inference. A prominent example is the Segment Anything Model (SAM), a promptable vision foundation model that can generate segmentation masks from user-provided points or bounding boxes [15]. Although SAM has demonstrated strong zero-shot performance in natural images, its performance in medical imaging has been more variable, particularly for specialized targets characterized by low contrast, complex anatomy, or poorly defined boundaries [16,17].

Medical-domain adaptations such as MedSAM and MedSAM2 have been developed to improve prompt-based segmentation in medical imaging [18,19]. In parallel, nnInteractive provides a promptable 3D interactive segmentation framework for volumetric medical images [20]. Together, these models reflect a growing interest in prompt-driven medical foundation models for image segmentation. However, their effectiveness against highly disease-specific targets, such as pancreatic tumors, remains uncertain, particularly when tumor boundaries are ill-defined and local anatomy is complex. Prior work in specialized medical segmentation tasks has shown that applying foundation models without task-specific adaptation can be suboptimal, whereas fine-tuning can substantially improve performance and generalization [21]. Therefore, an important open question is whether medical foundation models are sufficient for pancreatic tumor segmentation on CT or whether target-cohort adaptation of a SAM-based framework is necessary for reliable tumor delineation.

To address this question, we extended our previously developed Tumor-Adaptive Guidance for SAM (TAGS), a SAM-based 3D framework for volumetric pancreatic tumor segmentation on CT, to a large multicenter cohort [22]. We benchmarked two medical foundation model baselines, nnInteractive and MedSAM2 [19,20], against TAGS-based approaches comprising zero-shot TAGS and two fine-tuned TAGS variants. This comparison assessed whether task-specific adaptation improves pancreatic tumor delineation compared with direct application of broadly trained foundation models. To facilitate future research in this area, we additionally curated and publicly released a multicenter pancreatic tumor CT segmentation dataset containing 500 CECT examinations with corresponding pancreas and tumor annotations.

## 2. Methods

### 2.1. Study Design and Patient Cohorts

This retrospective multicenter study was approved by the coordinating Institutional Review Board (IRB) and by the ethics committee at each contributing institution, with waiver of informed consent. Four independent PDAC cohorts were assembled from participating centers: Center A, Center B, Center C, and Center D. Across all cohorts, PDAC was pathologically confirmed in accordance with the 2019 World Health Organization (WHO) Classification of Tumors of the Digestive System. A total of 500 CECT examinations acquired between 2015 and 2025 were included. The final dataset consisted of de-identified CT images with corresponding expert pancreas and tumor segmentation masks.

### 2.2. CT Acquisition and Reference Annotation

CECT examinations were acquired using multidetector CT scanners from multiple vendors in accordance with institutional clinical protocols. The majority of examinations (>95%) were acquired in the late arterial phase, with injection protocols following institutional practice. The images were retrospectively collected from the Picture Archiving and Communication Systems (PACS) of the participating institutions. All images were exported in Digital Imaging and Communications in Medicine (DICOM) format and converted to Neuroimaging Informatics Technology Initiative (NIfTI) format for analysis. Pancreas and pancreatic tumor segmentations were performed manually in 3D Slicer (version 5.10) by one of two abdominal radiologists, each with more than 5 years of abdominal radiology experience [23]. Each CECT examination was segmented by a single radiologist rather than by consensus. All segmentations were subsequently reviewed by senior abdominal radiologists with at least 15 years of experience, who directly edited any masks requiring correction to ensure annotation accuracy and consistency.

### 2.3. Inter-Observer Agreement and Intra-Observer Reliability for Segmentation

We calculated intra- and inter-observer agreements using the Dice Similarity Coefficient (DSC), the 95th-percentile Hausdorff Distance (HD95), and the Normalized Surface Dice (NSD) at 5 mm. To assess inter-observer reliability, 30 CT examinations were randomly selected and independently segmented by the two abdominal radiologists. These cases were selected from examinations that had not been previously segmented by either radiologist. These 30 examinations are included among the 500 cases in the study cohort; the second segmentation was generated solely for reliability assessment and was not used as the reference standard. The inter-observer analysis yielded a mean DSC of 0.742, an HD95 of 5.32 mm, and an NSD of 0.923. To assess intra-observer reliability, one radiologist repeated tumor segmentation on 30 randomly selected CT examinations following a three-week washout period. The intra-observer analysis yielded a mean DSC of 0.838, an HD95 of 4.51 mm, and an NSD of 0.956.

### 2.4. Segmentation Models

We compared five segmentation models: two directly applied promptable foundation models and three TAGS configurations representing increasing levels of task-specific adaptation.

#### 2.4.1. nnInteractive

This interactive 3D medical segmentation foundation model is built on the nnU-Net framework and trained on a large multicenter corpus of volumetric scans [20,24]. It accepts point, scribble, lasso, and box prompts and refines its prediction using an AutoZoom mechanism that iteratively refocuses the network on the prompted region. We used the official nnInteractive checkpoint without modification and supplied a single foreground point located within the tumor. This constitutes a standardized single-point, single-pass protocol adopted for cross-method comparability and is not intended to characterize nnInteractive’s full interactive performance under its intended iterative workflow.

#### 2.4.2. MedSAM2

This model adapts SAM 2 to volumetric medical imaging by treating a CT volume as a sequence of 2D slices and propagating segmentation across the depth axis via SAM 2’s memory-attention module [19,25]. We used the publicly released MedSAM2 checkpoint (MedSAM2_latest) with the sam2.1_hiera_t512 configuration. Consistent with the inference protocol described in the original MedSAM2 paper, a bounding box was placed on the central slice of the tumor, and the resulting mask was propagated bidirectionally toward both ends of the volume. We note that MedSAM2’s video-style memory mechanism is designed around a spatially anchored prompt on a key frame, and box prompts, rather than single points, are the recommended interaction modality for 3D propagation in the original work.

#### 2.4.3. TAGS (Zero-Shot)

**Figure 1:**
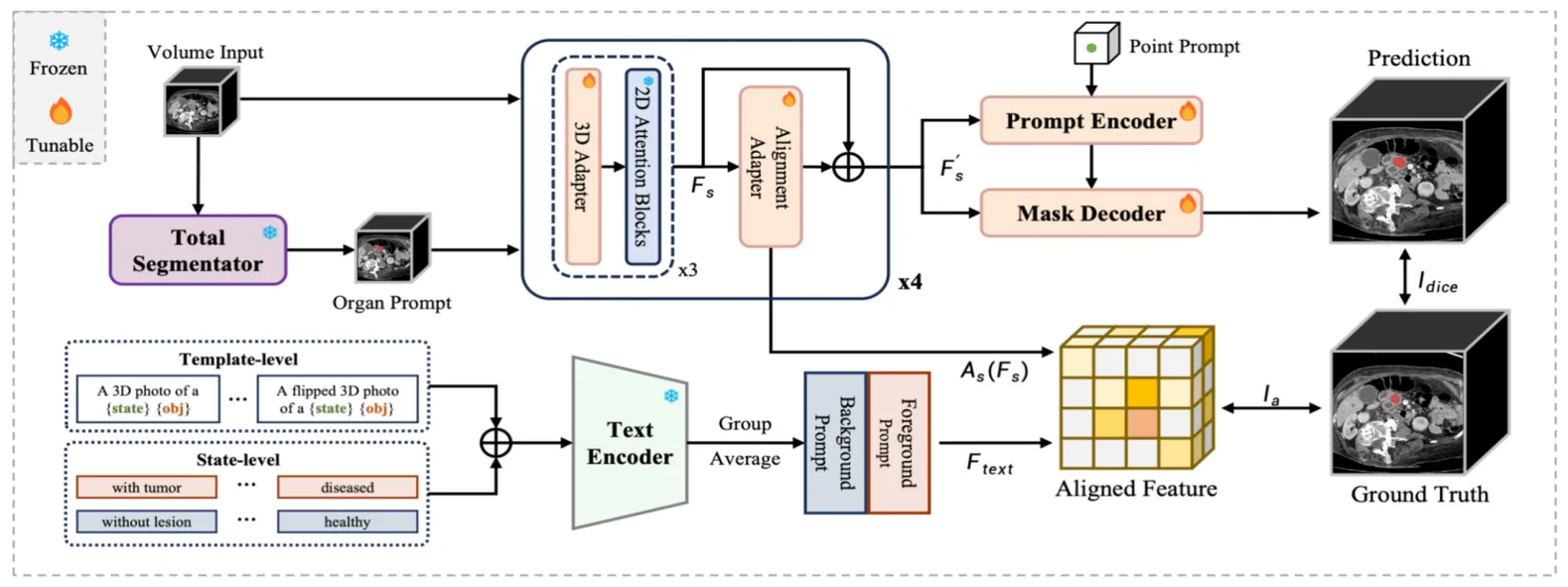
The overall framework of the proposed TAGS model. TAGS incorporates three complementary prompts: (1) an annotation-free organ prompt generated by a pretrained TotalSegmentator model to provide pancreas localization; (2) dual-category text prompts representing foreground (tumor) and background classes, encoded by the CLIP text encoder and integrated through stage-wise alignment adapters; and (3) interactive 3D point prompts providing sparse tumor localization cues. The organ prompt is input together with the CT volume, while semantic information from the text prompts is fused with image features through the alignment adapters. The resulting features are combined with the point prompt and processed by the mask decoder to predict the tumor segmentation.

TAGS is our previously published 2D-to-3D adaptation of SAM for volumetric tumor segmentation [15,22]. The overall framework is illustrated in Figure 1. The model combines four key components: a frozen SAM-B image encoder with stage-wise 3D adapters, an annotation-free organ prompt, a dual-category CLIP text prompt, and interactive 3D point prompts [26].

For anatomical guidance, the annotation-free organ prompt is generated using a pretrained TotalSegmentator model, which automatically segments the pancreas without requiring manual organ annotations from the target dataset [27]. The resulting pancreas mask is used as a spatial prior to guide the model toward the anatomical region of interest without requiring pseudo-label generation or additional manual annotation.

For semantic guidance, TAGS employs dual-category text prompts that represent the foreground (pancreatic tumor) and background (non-tumor tissue) classes. These text descriptions are encoded using the CLIP text encoder to generate category-specific text embeddings. The embeddings are subsequently integrated into the stage-wise alignment adapters, which align intermediate visual features extracted from the image encoder with the corresponding semantic representations, thereby enhancing tumor-background discrimination during feature extraction.

For interactive guidance, 3D point prompts provide sparse localization cues for segmentation. During training, point prompts are simulated from the ground-truth tumor annotations by randomly sampling foreground points within the tumor region. During inference, a single foreground point located within the tumor is provided as user input. The point prompt is encoded by the SAM prompt encoder and merged with the image features before mask prediction.

During inference, only the organ prompt, alignment adapters, and a single foreground point prompt are retained. For the zero-shot setting, we used the TAGS weights trained on the Medical Segmentation Decathlon (MSD) Pancreas dataset and applied them directly to the four target cohorts without any further adaptation. This setting evaluates the ability of a tumor-specific foundation model to generalize across institutions, scanners, and patient populations.

#### 2.4.4. SAM-TAGS

This variant fine-tunes the TAGS architecture from the original SAM-B weights on the combined training splits of the four target cohorts, keeping the SAM-B image encoder frozen and updating only the adapter, prompt encoder, and decoder parameters, as in the original TAGS recipe. This setting represents adaptation of the TAGS framework to a new clinical cohort without prior pancreas-specific pretraining.

#### 2.4.5. MSD-TAGS

This model uses the same training recipe as SAM-TAGS but initializes from the MSD-Pancreas TAGS checkpoint rather than from the original SAM-B weights. By comparing MSD-TAGS with SAM-TAGS, we evaluated whether a pancreas-specific checkpoint provides a more effective initialization for target-cohort fine-tuning.

### 2.5. Training and Inference Protocol

Prior to model development, all volumes were resampled to an isotropic spacing of 1 × 1 × 1 mm^3^, clipped to a fixed Hounsfield-unit window of [−39, 204] HU, specified a priori and applied identically to all volumes, and min–max normalized to [0,1]. For patch-based methods, a fixed input size of 128 × 128 × 128 voxels centered on the prompt was used during inference. All results were obtained using patient-level five-fold cross-validation. The same cross-validation splits were used for all evaluated methods to ensure a fair comparison. In each fold, patients were randomly partitioned into training (70%), validation (10%), and testing (20%) subsets at the patient level, with no overlap between subsets. In addition to pooled cross-validation, a leave-one-center-out analysis was performed in which models were trained on three centers and evaluated on the remaining center, repeated for each of the four institutions.

Within each training fold, SAM-TAGS and MSD-TAGS were trained using the AdamW optimizer with an initial learning rate of 1 × 10^−4^ and a batch size of one for 200 epochs on a single NVIDIA A100 (80 GB) GPU. The training objective combined a Dice loss on the predicted tumor mask with the multi-modal alignment loss defined in the original TAGS work. Data augmentation followed the original TAGS protocol and included random rotation, flipping, intensity shift, and random zoom. Model selection was based on the best validation Dice score.

At inference, each method was run with its native pipeline on the full volume: a single 128^3^ patch centered on the point prompt for the TAGS variants, AutoZoom for nnInteractive, and bidirectional slice propagation for MedSAM2. With the exception of MedSAM2, which requires a bounding-box prompt as discussed in Section 2.4.2, all methods received a single foreground point sampled from the reference tumor mask. We emphasize that TAGS additionally uses a TotalSegmentator-derived pancreas mask and dual-category text embeddings as integral components of its design; the comparison is therefore between complete systems as deployed rather than under matched prompting conditions. SAM-TAGS and MSD-TAGS share the identical prompt configuration and differ only in initialization, isolating the effect of the pretrained checkpoint.

### 2.6. Evaluation Metrics

Segmentation accuracy was quantified using the DSC for volumetric overlap and the NSD at a 5 mm tolerance for boundary agreement. Both metrics were computed in the original image space after resampling predictions back to the native voxel grid.

To characterize computational efficiency, we additionally report the total number of model parameters (M), the forward FLOPs (TFLOPs) per volume, and the wall-clock inference time per volume on a single A100 GPU. Inference time was averaged over 50 timed runs following 10 warm-up runs, with CUDA synchronization before and after each measurement. All methods were evaluated in FP32 precision without autocast. For MedSAM2, FLOPs and inference time are reported per volume by aggregating across the 128 slices of the propagation sequence, reflecting the computational cost of its native per-frame inference paradigm.

The efficiency figures reflect different inference paradigms across methods and are not directly comparable on a like-for-like basis. For the TAGS variants, the per-volume cost corresponds to a single 128^3^ patch. Because TAGS additionally requires a pancreas organ prompt at inference, we report the end-to-end TAGS cost, including the TotalSegmentator stage (v2.11.0, total task with pancreas ROI subset, multilabel output, GPU inference), for which only wall-clock time was measured (approximately 6.2 s per volume).

### 2.7. Statistical Analysis

Overall performance was summarized as the case-weighted mean across all cases, matching the per-case scores used for statistical testing; center-specific values were calculated within each institution. Performance distributions were summarized using mean ± standard deviation and median (interquartile range). For each method, 95% confidence intervals for mean DSC and NSD were obtained from 10,000 bootstrap resamples over cases. Paired comparisons between MSD-TAGS and each of the other evaluated methods were performed using two-sided Wilcoxon signed-rank tests on per-case DSC and NSD scores. For each comparison, the median paired difference with a 95% confidence interval obtained from 10,000 bootstrap resamples and the matched-pairs rank-biserial correlation (*r*) as an effect-size measure were reported. Exact *p*-values were adjusted for eight comparisons (four comparators across two metrics) using the Holm–Bonferroni method, with statistical significance defined as an adjusted two-sided *p*-value < 0.05. Failure rates were additionally summarized as the proportion of cases with DSC < 0.50 and DSC < 0.20. All statistical analyses were performed in Python (version 3.9.23) using the SciPy library (version 1.9.1).

## 3. Results

### 3.1. Cohort Characteristics

This study included 500 CECT scans from 500 patients with PDAC from four independent institutions: Center A, Center B, Center C, and Center D. The mean patient age ranged from 63.1 ± 9.6 to 68.9 ± 9.8 years across the participating institutions. Male patients comprised 49.5% to 70.3% of each cohort. Tumors spanning T1–T4 stages were represented across the multicenter cohort. The mean tumor size ranged from 4.7 ± 2.3 cm to 6.2 ± 2.8 cm. Median in-plane spacing ranged from 0.76 mm (IQR: 0.70–0.81) to 0.89 mm (IQR: 0.82–0.96), while median slice thickness ranged from 1.00 mm (IQR: 1.00–1.00) to 3.00 mm (IQR: 2.50–4.00) across the four cohorts. Scanner vendors also varied across institutions, reflecting the multicenter nature of the dataset. Detailed demographic, tumor, and imaging characteristics are summarized in Table 1.

### 3.2. Overall Segmentation Performance

**Figure 2:**
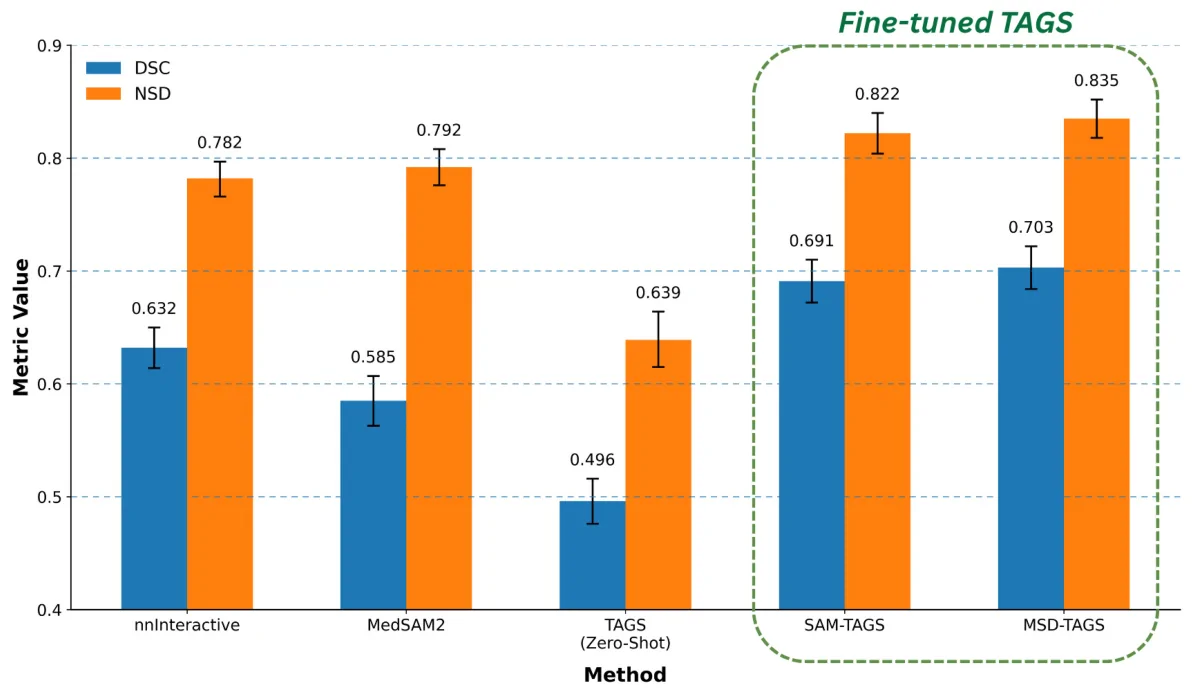
Bar plots showing the mean DSC and NSD for nnInteractive, MedSAM2, TAGS (Zero-Shot), SAM-TAGS, and MSD-TAGS. Values are case-weighted means across all 500 cases; error bars indicate 95% confidence intervals of the mean. The dashed box groups the two fine-tuned TAGS variants. The y-axis is truncated to improve visibility of between-method differences.

**Figure 3:**
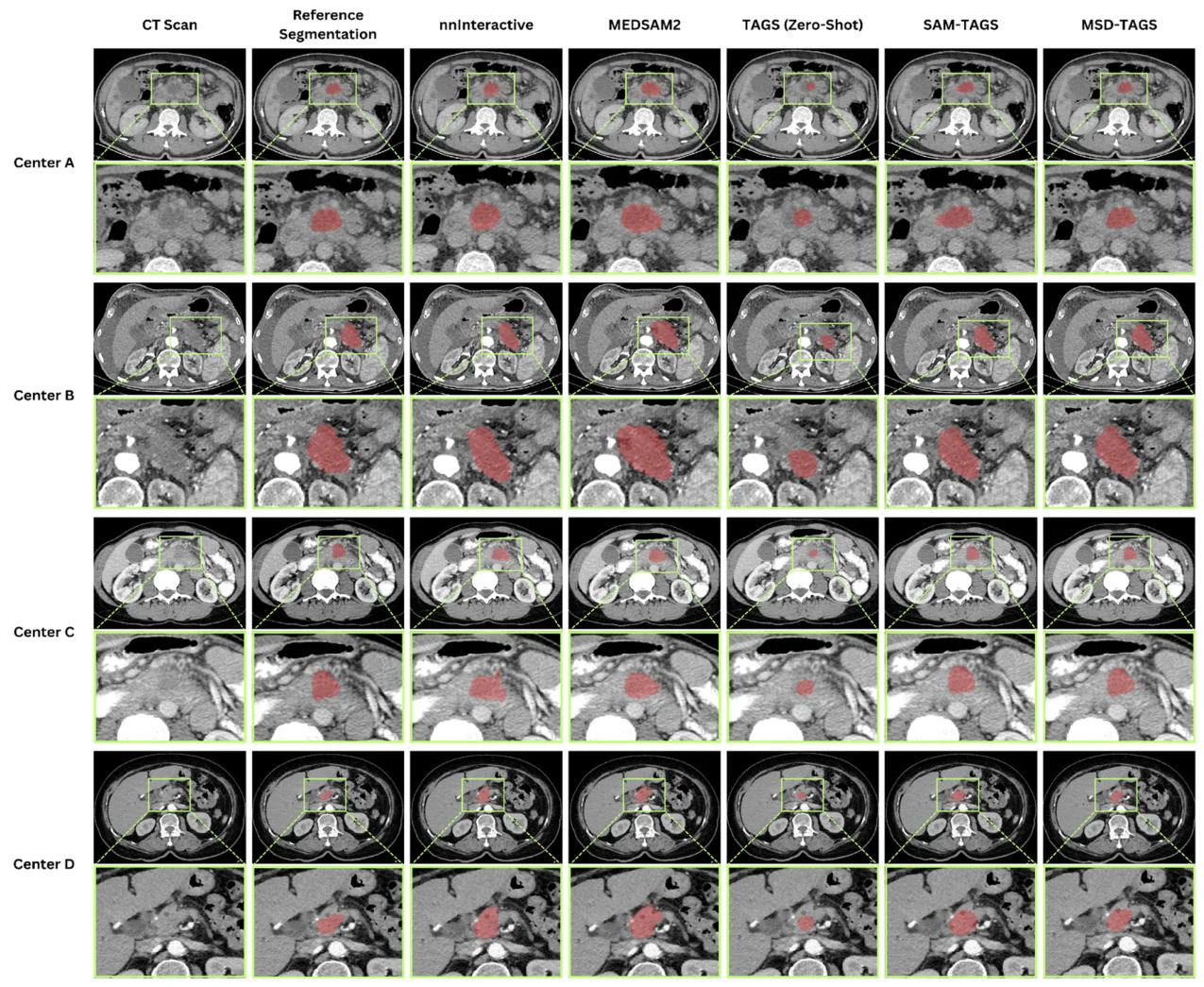
Representative pancreatic tumor segmentation outputs generated by the evaluated methods across Centers A–D. Columns show the original contrast-enhanced CT image, reference manual segmentation, nnInteractive, MedSAM2, TAGS (Zero-Shot), SAM-TAGS, and MSD-TAGS predictions. Tumor segmentation masks are displayed as red overlays. Green boxes indicate the region enlarged in the corresponding zoomed inset.

Quantitative segmentation results are summarized in Table 2. Among all evaluated methods, the fine-tuned TAGS variants consistently outperformed the directly applied models. MSD-TAGS achieved the highest overall performance, with a mean DSC of 0.703 ± 0.217 (95% CI 0.684–0.722) and a mean NSD of 0.835 ± 0.197 (95% CI 0.818–0.852), followed by SAM-TAGS (0.691 and 0.822). Among the directly applied foundation models, nnInteractive achieved a mean DSC of 0.632 and a mean NSD of 0.782, while MedSAM2 achieved 0.585 and 0.792, respectively. MSD-TAGS achieved significantly higher DSC and NSD than both nnInteractive and MedSAM2 (all Holm-adjusted *p* < 0.001; Supplementary Table S1). The proportion of cases with DSC below 0.50 was 15.8% for MSD-TAGS, 15.8% for SAM-TAGS, 22.6% for nnInteractive, 33.8% for MedSAM2, and 47.0% for TAGS (Zero-Shot); corresponding proportions below 0.20 were 4.8%, 5.0%, 5.6%, 9.0%, and 12.6%, respectively. Figure 2 summarizes the mean DSC and NSD achieved by each method across all 500 cases, while representative segmentation outputs produced by each evaluated method are shown in Figure 3.

### 3.3. Effect of Task-Specific Adaptation

A progressive increase in segmentation performance was observed as task-specific adaptation increased. Direct application of TAGS (Zero-Shot) yielded a mean DSC of 0.496 and an NSD of 0.639. Fine-tuning on the target cohorts raised these values to 0.691 and 0.822 for SAM-TAGS, and initialization from the MSD-Pancreas checkpoint raised them further to 0.703 and 0.835 for MSD-TAGS. Both fine-tuned variants also reduced the proportion of cases with DSC below 0.50 from 47.0% to 15.8%. The improvement of MSD-TAGS over SAM-TAGS was statistically significant but small in absolute terms (median paired DSC difference 0.013, 95% CI 0.010–0.016; rank-biserial *r* = 0.314; Holm-adjusted *p* < 0.001), whereas the corresponding difference relative to TAGS (Zero-Shot) was substantially larger (0.162, 95% CI 0.138–0.197; Supplementary Table S1).

### 3.4. Center-Wise and Leave-One-Center-Out Performance

**Figure 4:**
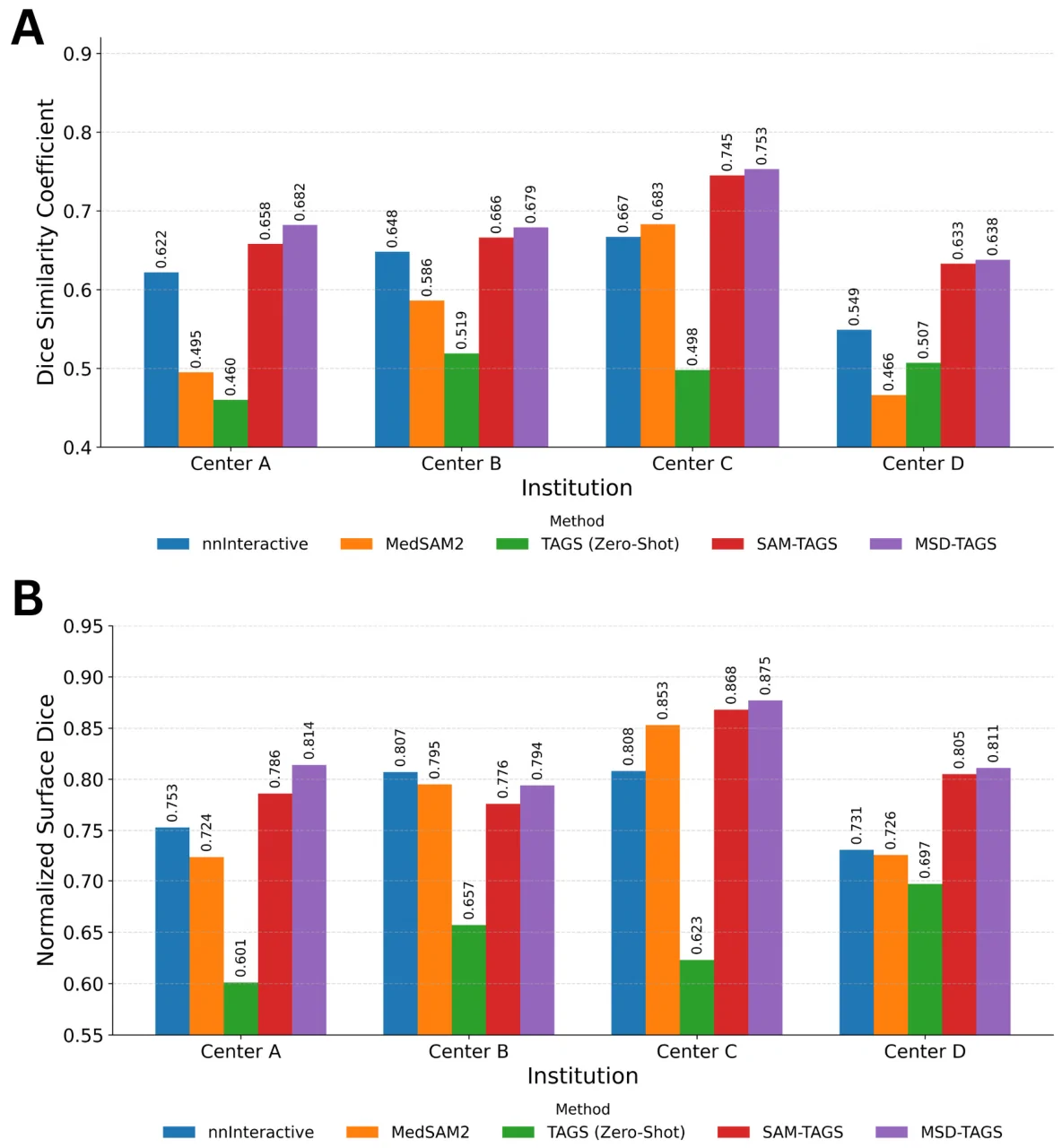
Center-wise segmentation performance of all evaluated methods across four participating institutions. (**A**) DSC and (**B**) NSD are shown for each center. The y-axes are truncated to improve visibility of between-method differences.

Center-wise segmentation results are presented in Table 3. MSD-TAGS achieved the highest DSC across all four participating institutions: Center A (0.682), Center B (0.679), Center C (0.753), and Center D (0.638). For boundary agreement, MSD-TAGS achieved the highest NSD at three of the four institutions: Center A (0.814), Center C (0.875), and Center D (0.811). At Center B, nnInteractive achieved the highest NSD (0.807), followed by MedSAM2 (0.795) and MSD-TAGS (0.794). Across all institutions, both fine-tuned TAGS variants consistently outperformed TAGS (Zero-Shot) in both metrics (Figure 4). In the additional leave-one-center-out (LOCO) analysis, mean DSC and NSD decreased from 0.691 and 0.822 to 0.602 and 0.736 for SAM-TAGS, and from 0.703 and 0.835 to 0.678 and 0.811 for MSD-TAGS. The reduction was substantially smaller for MSD-TAGS than for SAM-TAGS (DSC: 0.025 versus 0.089; NSD: 0.024 versus 0.086). Center-specific LOCO results are provided in Supplementary Table S2.

### 3.5. Computational Efficiency

Model complexity and computational performance are summarized in Table 4. The TAGS-based models contained 126.16 million parameters, required 7.455 TFLOPs, and achieved an average segmentation-network inference time of 0.682 s per volume. Because TAGS additionally requires a pancreas organ prompt at inference, we report the end-to-end cost including the TotalSegmentator stage (approximately 6.2 s per volume), giving a total of approximately 6.9 s per volume. nnInteractive achieved the shortest inference time (0.255 s) but required the highest per-volume computational cost (17.996 TFLOPs). MedSAM2 contained the fewest parameters (38.96 million) but required 9.237 TFLOPs with an average inference time of 4.968 s per volume; although it has the fewest parameters, its video-style memory mechanism propagates across 128 slices with per-frame memory attention, so its computational cost scales with sequence length rather than parameter count. Because these methods use different inference paradigms (a single 128^3^ patch for TAGS, AutoZoom for nnInteractive, and 128-slice propagation for MedSAM2), the reported FLOPs and times reflect different pipelines and are not directly comparable on a like-for-like basis.

## 4. Discussion

This multicenter study evaluated five segmentation approaches on a heterogeneous dataset of 500 pancreatic tumor CT scans from four centers. Our findings demonstrate that task-adapted TAGS variants outperformed two publicly released promptable models applied without task-specific adaptation, supporting the importance of disease- and task-specific adaptation for pancreatic tumor segmentation. Among all evaluated methods, MSD-TAGS achieved the highest mean performance, with a mean DSC of 0.703 for volumetric overlap and a mean NSD of 0.835 for boundary agreement, while SAM-TAGS also performed strongly with a mean DSC of 0.691 and NSD of 0.822. The mean DSC for MSD-TAGS (0.703) fell modestly below the inter-observer DSC of 0.742, and the mean NSD (0.835) fell further below the inter-observer NSD of 0.923, indicating that volumetric and boundary agreement come close to, but do not yet reach, reader-level reproducibility. These findings are particularly relevant in multicenter pancreatic tumor imaging. Substantial anatomic variability of the pancreas, poorly defined or isoattenuating tumor appearance, and cross-institutional imaging heterogeneity can limit model generalizability across institutions [28,29].

The TAGS-based comparisons highlight the value of adaptation. Fine-tuned TAGS variants outperformed TAGS (Zero-Shot) in both volumetric overlap and boundary agreement, suggesting that applying TAGS without target-cohort fine-tuning can be insufficient for heterogeneous, multicenter cohorts. The additional improvement of MSD-TAGS over SAM-TAGS suggests that initialization from a pancreas- and tumor-relevant checkpoint may provide a more effective starting point for target-cohort fine-tuning. This advantage should not be attributed to pancreas-specific pretraining alone, as the two models differ in both initialization and prior training data exposure. Although this improvement was modest relative to inter-observer variability, whether it translates into meaningful differences for treatment planning, surgical assessment, or tumor burden quantification requires task-specific clinical validation. These findings align with recent pancreatic tumor MRI segmentation work showing that multi-stage fine-tuning, from general anatomical pretraining to pancreas-specific lesion data and target MRI modalities, can improve performance in limited-data settings [30]. Similarly, prior SAM-based medical segmentation studies have shown that appropriate fine-tuning can substantially improve foundation model performance, making these models competitive for medical segmentation tasks [31].

TAGS (Zero-Shot) was the lowest-performing method, with a mean DSC of 0.496 and NSD of 0.639, performing worse than both general-purpose promptable baselines. Despite tumor-specific pretraining, TAGS (Zero-Shot) transferred less effectively to this heterogeneous multicenter cohort than models pretrained on substantially larger and more diverse corpora. This finding suggests that tumor-specific pretraining alone may not provide robust cross-institutional generalization without adaptation to the target cohort.

Compared with nnInteractive and MedSAM2, the fine-tuned TAGS variants achieved greater overall tumor overlap. Although both models served as foundation model baselines, their mean DSC values remained lower at 0.632 and 0.585, respectively. For surface boundary agreement, the fine-tuned TAGS variants also showed higher mean NSD values overall, although this pattern was not uniform across all centers. Similar findings were reported by Nguyen et al., who showed that direct application of LiteMedSAM performed poorly, whereas task-specific fine-tuning improved both overlap and Hausdorff distance metrics [32]. Thus, medical foundation models can serve as useful starting points, but task-specific adaptation appears important for strong performance in disease-specific segmentation tasks.

In the center-held-out analysis, performance declined at unseen institutions, reflecting a cross-center distribution shift. The reduction was substantially smaller for MSD-TAGS than for SAM-TAGS (DSC: 0.025 versus 0.089), suggesting greater robustness of MSD-TAGS at unseen institutions. Notably, MSD-TAGS achieved a higher DSC at entirely withheld centers (0.678) than nnInteractive (0.632) and MedSAM2 (0.585) achieved across the full cohort. Pooled multicenter development nonetheless yielded higher performance by exposing models to institutional heterogeneity during training.

Prior pancreatic tumor segmentation studies have often relied on specialized architectures, including cascaded pancreas-to-tumor pipelines, localization-guided frameworks, multi-scale feature extraction, and attention mechanisms [33,34,35,36]. The relatively lower performance of directly applied foundation models in our cohort underscores the technical difficulty of pancreatic tumor segmentation. These findings suggest that task-adapted SAM-based frameworks may provide a practical alternative to developing entirely new task-specific segmentation architectures.

Practical efficiency is an important consideration when applying volumetric models across large clinical imaging cohorts. Therefore, model utility should be assessed using computational benchmarks alongside segmentation performance. MedSAM2 had the smallest parameter count but required the longest segmentation-network inference time (4.968 s per volume), with lower DSC and NSD values than the fine-tuned TAGS variants. In contrast, nnInteractive provided the fastest inference (0.255 s per volume) and achieved a higher mean DSC than MedSAM2, but its overall segmentation performance remained lower than that of SAM-TAGS and MSD-TAGS. Overall, although the fine-tuned TAGS variants had higher parameter counts, they achieved the strongest segmentation performance at the lowest segmentation-network FLOP count. We note, however, that TAGS operates as a localize-then-segment cascade whose end-to-end cost includes a preceding TotalSegmentator organ-localization stage, bringing total inference to approximately 6.9 s per volume; its principal inference cost therefore lies in this localization step rather than in the segmentation network. MSD-TAGS achieved a modest performance advantage over SAM-TAGS at the same segmentation-network cost.

Clinical applicability depends on factors beyond segmentation performance alone. Interaction time, re-prompting frequency, and radiologist workload were not assessed, and no downstream clinical endpoint was evaluated, so the clinical benefit for tasks such as surgical planning, radiotherapy planning, vessel invasion assessment, or recurrence prediction cannot be quantified from our data. Fine-tuning reduced the proportion of cases with DSC below 0.50 from 47.0% to 15.8%, and these are the cases most likely to require substantial manual correction rather than minor editing. Boundary agreement for MSD-TAGS (NSD 0.835) approached but did not reach the inter-observer reference of 0.923, and the difference between the two fine-tuned variants was small against that variability. As with other automated segmentation methods, clinical use would require radiologist review. Uncertainty estimation, which could help triage cases for closer inspection, was not evaluated here and remains a direction for future work.

An additional contribution of this study is the public release of a multicenter pancreatic tumor CT segmentation dataset consisting of 500 CECT examinations with corresponding pancreas and tumor segmentation annotations. Publicly available multicenter pancreatic tumor CT segmentation datasets remain limited, particularly at this scale and in terms of multicenter representation. By releasing the imaging data and segmentation masks, we aim to facilitate reproducible benchmarking, studies of foundation model adaptation, and the further development of automated pancreatic cancer imaging tools.

This study has some limitations that should be considered when interpreting the results. First, the retrospective design could introduce selection bias related to case availability and imaging quality. Second, T stage was unavailable in 21.5% to 67.7% of cases across centers, with missingness systematically associated with center rather than being random; stage-stratified analyses were therefore not pursued. Third, reliance on manual 3D tumor segmentation by radiologists may limit reproducibility and scalability because delineation remains susceptible to operator variability, particularly for ill-defined or isoattenuating pancreatic lesions. Although expert oversight and reproducibility assessments were performed, some degree of variability is inevitable. Fourth, although a standardized prompting protocol was used, prompt-based segmentation can vary with prompt placement and user interaction. This issue is further complicated by model-specific prompting requirements: MedSAM2 was evaluated with bounding-box prompts consistent with its native inference protocol, whereas the other models used point-based prompts. Therefore, performance comparisons should be interpreted in light of these methodological differences. Fifth, segmentation performance was evaluated against expert reference annotations; whether the observed differences alter downstream clinical determinations was not assessed. Finally, this study was designed to isolate the effect of target-cohort adaptation within a promptable foundation-model framework rather than to re-benchmark the TAGS architecture against conventional supervised networks, which was reported previously on a separate dataset [22]. Future studies should validate these findings in larger, more diverse datasets and evaluate performance against downstream clinical endpoints. Despite these limitations, the superior performance of the fine-tuned TAGS variants supports the value of task-specific adaptation for pancreatic tumor segmentation.

## 5. Conclusions

This multicenter feasibility study demonstrated that task-specific adaptation improved pancreatic tumor segmentation with foundation models. While general-purpose interactive and medical foundation models provided useful baselines, fine-tuned TAGS variants consistently achieved superior segmentation performance across the multicenter cohort. The progressive improvements observed with increasing adaptation suggest that organ-specific initialization and target-cohort fine-tuning improve performance in disease-specific segmentation tasks. These findings support the growing role of task-adapted foundation models in pancreatic cancer imaging and lay the groundwork for foundation model-based segmentation workflows, which warrant further validation before clinical translation.

## Figures and Tables

**Table 1:** Baseline demographic, tumor characteristics, and imaging parameters of the cohorts.

Characteristic		Center A (*n* = 101)	Center B (*n* = 93)	Center C (*n* = 210)	Center D (*n* = 96)
**Demographics**					
Age, years (±SD)		63.8 ± 9.6	67.7 ± 10.8	68.9 ± 9.8	63.1 ± 9.6
Male sex, *n* (%)		71 (70.3)	53 (57.0)	104 (49.5)	60 (62.5)
Female sex, *n* (%)		29 (28.7)	37 (39.8)	106 (50.5)	36 (37.5)
Unknown, *n* (%)		1 (1.0)	3 (3.2)	-	-
**Tumor Characteristics**					
Tumor size, cm (±SD)		5.6 ± 2.5	4.9 ± 2.4	6.2 ± 2.8	4.7 ± 2.3
T1, *n* (%)		4 (4.0)	6 (6.5)	9 (4.3)	5 (5.2)
T2, *n* (%)		27 (26.7)	30 (32.3)	42 (20.0)	11 (11.5)
T3, *n* (%)		10 (9.9)	21 (22.6)	28 (13.3)	15 (15.6)
T4, *n* (%)		37 (36.6)	16 (17.2)	30 (14.3)	-
Unknown, *n* (%)		23 (22.8)	20 (21.5)	101 (48.1)	65 (67.7)
**Imaging Parameters**					
In-plane spacing, mm (IQR)		0.85 (0.78–0.92)	0.81 (0.76–0.87)	0.76 (0.70–0.81)	0.89 (0.82–0.96)
Slice thickness, mm (IQR)		1.00 (1.00–1.25)	1.00 (1.00–1.00)	3.00 (2.50–4.00)	2.00 (1.00–3.00)
Scanner vendor, *n* (%)	Canon	46 (45.5)	-	3 (1.4)	-
	GE	45 (44.6)	2 (2.2)	37 (17.6)	9 (9.4)
	Philips	4 (4.0)	1 (1.1)	1 (0.5)	-
	Siemens	2 (2.0)	90 (96.8)	95 (45.2)	87 (90.6)
	Toshiba	4 (4.0)	-	74 (35.2)	-

Data are presented as mean ± standard deviation, median (interquartile range), or *n* (%), as appropriate. Dashes indicate no cases in the given category.

**Table 2:** Segmentation performance across models.

	DSC			NSD		
Method	Mean ± SD	95% CI	Median (IQR)	Mean ± SD	95% CI	Median (IQR)
nnInteractive	0.632 ± 0.207	0.614–0.650	0.686 (0.520–0.788)	0.782 ± 0.180	0.766–0.797	0.819 (0.678–0.925)
MedSAM2	0.585 ± 0.249	0.563–0.607	0.661 (0.397–0.795)	0.792 ± 0.185	0.776–0.808	0.822 (0.672–0.944)
TAGS (Zero-Shot)	0.496 ± 0.229	0.476–0.516	0.526 (0.339–0.681)	0.639 ± 0.281	0.615–0.664	0.681 (0.422–0.890)
SAM-TAGS	0.691 ± 0.214	0.672–0.710	0.768 (0.612–0.841)	0.822 ± 0.205	0.804–0.840	0.901 (0.769–0.965)
MSD-TAGS	**0.703 ± 0.217**	**0.684–0.722**	**0.780 (0.632–0.856)**	**0.835 ± 0.197**	**0.818–0.852**	**0.909 (0.776–0.976)**

Bold values indicate the results of the best-performing method for each metric.

**Table 3:** Segmentation performance across cohorts.

	Center A		Center B		Center C		Center D	
Method	DSC	NSD	DSC	NSD	DSC	NSD	DSC	NSD
nnInteractive	0.622 ± 0.186	0.753 ± 0.179	0.648 ± 0.235	**0.807 ± 0.184**	0.667 ± 0.181	0.808 ± 0.159	0.549 ± 0.231	0.731 ± 0.205
MedSAM2	0.495 ± 0.254	0.724 ± 0.181	0.586 ± 0.262	0.795 ± 0.193	0.683 ± 0.205	0.853 ± 0.155	0.466 ± 0.232	0.726 ± 0.198
TAGS (Zero-Shot)	0.460 ± 0.243	0.601 ± 0.292	0.519 ± 0.238	0.657 ± 0.281	0.498 ± 0.219	0.623 ± 0.281	0.507 ± 0.227	0.697 ± 0.263
SAM-TAGS	0.658 ± 0.217	0.786 ± 0.204	0.666 ± 0.266	0.776 ± 0.261	0.745 ± 0.167	0.868 ± 0.157	0.633 ± 0.224	0.805 ± 0.219
MSD-TAGS	**0.682 ± 0.211**	**0.814 ± 0.188**	**0.679 ± 0.262**	0.794 ± 0.249	**0.753 ± 0.185**	**0.875 ± 0.167**	**0.638 ± 0.216**	**0.811 ± 0.198**

Bold values indicate the results of the best-performing method for each metric.

**Table 4:** Computational efficiency of evaluated models.

Method	Parameters (M)	TFLOPs	Inference Time (s)
nnInteractive	102.35	17.996	0.255
MedSAM2	38.96	9.237	4.968
TAGS (Zero-Shot)	126.16	7.455	0.682 *
SAM-TAGS	126.16	7.455	0.682 *
MSD-TAGS	126.16	7.455	0.682 *

* TAGS inference uses a single 128^3^ patch (one patch per volume); the reported time is for the segmentation network only. End-to-end TAGS inference additionally includes the TotalSegmentator organ-localization stage (~6.2 s per volume), giving approximately 6.9 s per volume in total. FLOPs and times reflect different inference paradigms across methods and are not directly comparable.

## Data Availability

The de-identified multicenter pancreatic tumor CT segmentation dataset, comprising CECT volumes with corresponding expert-annotated pancreas and tumor masks, is publicly available in the Open Science Framework repository at https://osf.io/g6rv5/ (accessed on 29 June 2026). Source code and documentation are available at https://nubagcilab.github.io/TAGS_feasibility/ (accessed on 29 June 2026).

## Supplementary Materials

The following supporting information can be downloaded at https://www.mdpi.com/article/10.3390/cancers18172836/s1, Table S1: Paired statistical comparisons of MSD-TAGS with comparator methods across all cases; Table S2: Segmentation performance across cohorts under leave-one-center-out and pooled cross-validation.
