# Supplementary material for: Multicenter Validation of Foundation Model Adaptation for Automated Pancreatic Tumor Delineation on CT Scans
Source: Cancers (Basel). 2026 Sep 1;18(17):2836. doi: 10.3390/cancers18172836 (PMC13564768; doi:10.3390/cancers18172836)
Supplement: Supplementary file 1 [file cancers-18-02836-s001.zip › cancers-4437871-supplementary.pdf]

Table S1. Paired statistical comparisons of MSD-TAGS with comparator methods across all cases

Comparator	Metric	Median Paired Difference (95% CI)	Rank-Biserial *r*	Exact *p*	Holm-Adjusted *p*
SAM-TAGS	**DSC**	0.013 (0.010–0.016)	0.314	1.21×10^{-9}	3.64×10^{-9}
	**NSD**	0.010 (0.007–0.015)	0.262	3.91×10^{-7}	3.91×10^{-7}
nnInteractive	**DSC**	0.069 (0.054–0.086)	0.482	9.92×10^{-21}	4.96×10^{-20}
	**NSD**	0.053 (0.030–0.072)	0.371	6.93×10^{-13}	2.77×10^{-12}
MedSAM2	**DSC**	0.085 (0.070–0.098)	0.585	8.27×10^{-30}	4.96×10^{-29}
	**NSD**	0.040 (0.025–0.056)	0.306	3.53×10^{-9}	7.07×10^{-9}
TAGS (Zero-Shot)	**DSC**	0.162 (0.138–0.197)	0.714	1.68×10^{-43}	1.34×10^{-42}
	**NSD**	0.149 (0.118–0.179)	0.618	5.34×10^{-33}	3.73×10^{-32}

Note: *Differences were calculated as MSD-TAGS minus the comparator using paired per-case scores from the pooled five-fold cross-validation across all 500 cases. Median paired differences are reported with 95% confidence intervals obtained from 10,000 bootstrap resamples. Statistical significance was assessed using two-sided Wilcoxon signed-rank tests. Effect sizes are reported as matched-pairs rank-biserial correlations (r). Exact p-values were adjusted for eight comparisons (four comparators across two metrics) using the Holm–Bonferroni method. Positive differences and positive rank-biserial correlations indicate better performance for MSD-TAGS.*

Table S2. Segmentation performance across cohorts under leave-one-center-out and pooled cross-validation.

	Center A		Center B		Center C		Center D		All Cases	
**Method**	**DSC**	**NSD**	**DSC**	**NSD**	**DSC**	**NSD**	**DSC**	**NSD**	**DSC**	**NSD**
nnInteractive	0.622 ± 0.186	0.753 ± 0.179	0.648 ± 0.235	0.807 ± 0.184	0.667 ± 0.181	0.808 ± 0.159	0.549 ± 0.231	0.731 ± 0.205	0.632 ± 0.207	0.782 ± 0.180
MedSAM2	0.495 ± 0.254	0.724 ± 0.181	0.586 ± 0.262	0.795 ± 0.193	0.683 ± 0.205	0.853 ± 0.155	0.466 ± 0.232	0.726 ± 0.198	0.585 ± 0.249	0.792 ± 0.185
TAGS (Zero-Shot)	0.460 ± 0.243	0.601 ± 0.292	0.519 ± 0.238	0.657 ± 0.281	0.498 ± 0.219	0.623 ± 0.281	0.507 ± 0.227	0.697 ± 0.263	0.496 ± 0.229	0.639 ± 0.281
**SAM-TAGS (LOCO)**	0.610 ± 0.195	0.751 ± 0.188	0.646 ± 0.276	0.766 ± 0.273	0.577 ± 0.221	0.691 ± 0.251	0.607 ± 0.215	0.789 ± 0.198	0.602 ± 0.227	0.736 ± 0.237
**MSD-TAGS (LOCO)**	0.648 ± 0.231	0.780 ± 0.212	0.655 ± 0.263	0.783 ± 0.258	0.718 ± 0.188	0.836 ± 0.190	0.643 ± 0.221	0.817 ± 0.213	0.678 ± 0.221	0.811 ± 0.214
**SAM-TAGS (Pooled CV)**	0.658 ± 0.217	0.786 ± 0.204	0.666 ± 0.266	0.776 ± 0.261	0.745 ± 0.167	0.868 ± 0.157	0.633 ± 0.224	0.805 ± 0.219	0.691 ± 0.214	0.822 ± 0.205
**MSD-TAGS (Pooled CV)**	0.682 ± 0.211	0.814 ± 0.188	0.679 ± 0.262	0.794 ± 0.249	0.753 ± 0.185	0.875 ± 0.167	0.638 ± 0.216	0.811 ± 0.198	0.703 ± 0.217	0.835 ± 0.197

Note: *LOCO = leave-one-center-out, in which the model was trained on three centers and evaluated on the withheld center. Pooled CV = pooled patient-level five-fold cross-validation. nnInteractive, MedSAM2, and TAGS (Zero-Shot) were applied without training on the study cohort and therefore have no leave-one-center-out counterpart; their values are identical under both evaluation designs. Values are mean ± SD.*
